# N-Acetyl-Serotonin Protects HepG2 Cells from Oxidative Stress Injury Induced by Hydrogen Peroxide

**DOI:** 10.1155/2014/310504

**Published:** 2014-06-12

**Authors:** Jiying Jiang, Shuna Yu, Zhengchen Jiang, Cuihong Liang, Wenbo Yu, Jin Li, Xiaodong Du, Hailiang Wang, Xianghong Gao, Xin Wang

**Affiliations:** ^1^Departments of Anatomy, Weifang Medical University, Weifang, Shandong 261053, China; ^2^Department of Neurosurgery, Brigham and Women's Hospital, Harvard Medical School, Boston, MA 02115, USA

## Abstract

Oxidative stress plays an important role in the pathogenesis of liver diseases. N-Acetyl-serotonin (NAS) has been reported to protect against oxidative damage, though the mechanisms by which NAS protects hepatocytes from oxidative stress remain unknown. To determine whether pretreatment with NAS could reduce hydrogen peroxide- (H_2_O_2_-) induced oxidative stress in HepG2 cells by inhibiting the mitochondrial apoptosis pathway, we investigated the H_2_O_2_-induced oxidative damage to HepG2 cells with or without NAS using MTT, Hoechst 33342, rhodamine 123, Terminal dUTP Nick End Labeling Assay (TUNEL), dihydrodichlorofluorescein (H2DCF), Annexin V and propidium iodide (PI) double staining, immunocytochemistry, and western blot. H_2_O_2_ produced dramatic injuries in HepG2 cells, represented by classical morphological changes of apoptosis, increased levels of malondialdehyde (MDA) and intracellular reactive oxygen species (ROS), decreased activity of superoxide dismutase (SOD), and increased activities of caspase-9 and caspase-3, release of cytochrome c (Cyt-C) and apoptosis-inducing factor (AIF) from mitochondria, and loss of membrane potential (ΔΨ*_m_*). NAS significantly inhibited H_2_O_2_-induced changes, indicating that it protected against H_2_O_2_-induced oxidative damage by reducing MDA levels and increasing SOD activity and that it protected the HepG2 cells from apoptosis through regulating the mitochondrial apoptosis pathway, involving inhibition of mitochondrial hyperpolarization, release of mitochondrial apoptogenic factors, and caspase activity.

## 1. Introduction

It is now well established that oxidative stress, characterized by a cellular imbalance in the production and elimination of reactive oxygen species (ROS), plays an important role in the pathogenesis of various liver disorders, including liver inflammation [1–3], hepatic cirrhosis [4, 5], hepatoma [6], and hepatic ischemia-reperfusion (I/R) injury [7–9]. Hydrogen peroxide (H_2_O_2_) is an important cause of oxidative injury because its half-life is longer than that of other reactive oxygen species and it can easily transform into a hydroxyl radical, one of the most destructive free radicals [10]. Moreover, it is generated from nearly all sources of oxidative stress and can diffuse freely in and out of many kinds of cells and tissues. Furthermore, H_2_O_2_ has been used by other investigators to induce oxidative stress in myocytes [11], HT22 cells [12], retinal pigment epithelium cells [13], and BEL-7402 cells [14–16]. Therefore, we chose H_2_O_2_ to induce HepG2 cells death in our study because it has been reported to trigger apoptosis in hepatocytes, the main cause of hepatocyte death during hepatic I/R injury [17–22].

A large body of evidence indicates that though ROS are generated mainly as by-products of mitochondrial respiration, they are themselves extremely susceptible to oxidative damage. Thus, a feed-forward loop is set up whereby ROS-mediated oxidative damage to mitochondria favors more ROS generation, creating a vicious cycle [23]. The overproduction of ROS leads to lipid peroxidation, damage to the mitochondrial membrane, release of mitochondrial apoptogenic factors into the cytoplasm, followed by caspase activation and finally cellular apoptosis [24, 25]. It is becoming clear that mitochondria play a critical part in cellular bioenergetics through production of ROS and control of cell death. Therefore, modulation of mitochondrial function by antioxidants has become a novel therapeutic strategy to prevent oxidative damage, which is a key mechanism leading to liver inflammation, hepatic cirrhosis, hepatoma, and hepatic I/R injury.

Growing evidence supports the contention that H_2_O_2_-induced cell injury may be prevented by antioxidants such as N-acetyl-serotonin (NAS) and Vitamin E [26–28]. NAS, the immediate precursor of melatonin in the tryptophan metabolic pathway in the pineal gland, is a free radical scavenger. NAS has recently been shown to have various biologic activities, including memory-facilitating [29], hypothermic [30], analgesic [31], circadian rhythm adjustment [32], antidepressant [32], antiaging [33], lowering blood pressure [28], and antioxidative effects [28]. As described in our recent reports, NAS protects against acute hepatic I/R injury in mice by reversing the imbalance between oxidants and antioxidants and inhibiting hepatocyte apoptosis via the intrinsic apoptotic pathway [34] and offers neuroprotection through inhibiting mitochondrial death pathways in experimental models of ischemic stroke [35]. However, the mechanism by which NAS protects hepatocytes from oxidative stress remains unknown. Here we investigated the effects of NAS on oxidative HepG2 cells damage induced by H_2_O_2_ and whether mitochondria-dependent apoptosis was involved in the cytoprotective effect of NAS.

## 2. Materials and Methods

### 2.1. Chemicals

AIF (apoptosis-inducing factor) antibody, NAS, and 2,7-dichlorodihydrofluorescein diacetate were obtained from Sigma (St. Louis, MO). Cleaved caspase-3, cleaved caspase-9, Cyt-C, and *β*-actin antibodies were purchased from Cell Signaling Technology, Inc. (Beverly, MA). Cox-IV antibodies came from BD PharMingen (San Diego, CA). Horseradish-conjugated secondary anti-mouse antibody and anti-rabbit antibody were purchased from Amersham Pharmacia Biotech (Piscataway, NJ). FITC-conjugated secondary antibodies were purchased from Jackson Immunoresearch (West Grove, PA). Hoechst 33342, rhodamine 123, and tetramethylrhodamine methyl ester (TMRM) were purchased from Life Technologies Corporation (Grand Island, NY). The enhanced chemiluminescence (ECL) system was purchased from Amersham Pharmacia Biotech (Piscataway, NJ). MDA and SOD kits were obtained from the NanJing JianCheng Bioengineering Institute (Nanjing, China). The TUNEL apoptosis kits came from Boster Biotechnology (Wuhan, China). Annexin V-FITC/propidium iodide (PI) Apoptosis Detection Kit was purchased from Roche (Germany).

### 2.2. Cell Lines and Induction of Cell Death

The human hepatoma cell line HepG2 was kindly provided by Dr. Shujuan Liang. Originally derived from a hepatocellular carcinoma biopsy, this cell line synthesizes nearly all human plasma proteins. With high degree of differentiation, HepG2 cells retain many biological characteristics of hepatocytes and could synthesize nearly all human plasma proteins. It has been widely used as a cellular model to investigate the oxidative injury of hepatocytes elicited by H_2_O_2_ [36–40], arachidonic acid and iron [41], hepatocytes toxicity induced by alcohol [42] or CYP2E1 [43], and hepatocellular injury by Hepatitis C virus [44]. HepG2 cells were cultured in a humidified atmosphere of 95% air plus 5% CO_2_ in a 37°C incubator in Dulbecco's modified Eagle's medium supplemented with 10% heat-inactivated fetal bovine serum, 100 *μ*M streptomycin, and 100 U/mL penicillin. Cell death was induced by exposure to H_2_O_2_ as previously demonstrated by others [45, 46]. In brief, cells were challenged with H_2_O_2_ with or without NAS for 6 hours. The morphology of HepG2 cell nuclei was analyzed with Hoechst 33342 and Annexin V-FITC/PI double staining.

### 2.3. Determination of Cytotoxicity by MTT Assay

The MTT assay is based on the principle that viable cells convert MTT into an insoluble formazan salt. The absorbance at 490 nm was read in an ELISA plate reader. Briefly, HepG2 cells were cultured in 96-well microtiter plates in a final volume of 100 *μ*L culture medium per well. After incubation for 24 hours at 37°C and 5% CO_2_, when a partial monolayer was formed, the supernatant was flicked off, washed once, 20 *μ*L of MTT (5 mg/mL) in PBS solution was added to each well, and then the plate was further incubated for 4 h. Most of the medium was removed and 100 *μ*L of DMSO was added into the wells to solubilize the crystals. Finally the optical density (OD) was measured by ELISA reader at wavelength of 490 nm. All cytotoxicity assays were performed in duplicate. The percentage growth inhibition was calculated using the following formula:

(1) Cell  viability  (%)  =the  absorbance  of  experimental  groupthe  absorbance  of  blank  untreated  group×100%.

### 2.4. MDA and SOD Assays

MDA and SOD in HepG2 cells were measured with commercial kits according to the manufacturer's recommendations as previously described [23]. MDA levels and SOD activity were expressed as nmol/mg protein and U/mg protein, respectively.

### 2.5. Intracellular ROS Measurement

ROS was monitored using dihydrodichlorofluorescein (H2DCF) cell-permeant probe according to the manufacturer's recommendations. Briefly, the cells from different groups were collected and incubated for 20 min in the dark at 37°C in PBS containing 10 *μ*M H2DCF. The levels of ROS in HepG2 cells were analyzed by measuring the mean fluorescence intensity (MFI) of DCF using a FACSCalibur flow cytometer (Becton-Dickinson, San Jose, CA, USA) with an excitation of 485 nm and emission of 530 nm. The results were expressed as the fold change of the percentage increase in DCF channel. The experiments were performed in triplicate and repeated three times.

### 2.6. Observation of Nuclear Damage by Hoechst 33342 Staining

HepG2 cells were exposed to 200 *μ*M H_2_O_2_ with or without NAS for 6 hours. The cells were then incubated with Hoechst 33342 (1 : 10,000) for 5 minutes, and changes to cell nuclei were observed by fluorescence microscope (OLYMPUS, BX53F).

### 2.7. Terminal dUTP Nick End Labeling Assay (TUNEL)

HepG2 cells were fixed in 4% paraformaldehyde, permeabilized with 0.05% Triton X-100, and then subjected to a TUNEL assay with commercial kits according to the manufacturer's recommendations as previously described [23]. TUNEL-positive cells were colored using diaminobenzidine (DAB) and counterstained with hematoxylin.

### 2.8. Annexin V-FITC/Propidium Iodide Double Staining as a Measure of Cell Apoptosis

Briefly, cells were trypsinized following a wash in PBS and resuspended in 400 *μ*L 1x binding buffer. Then 5 *μ*L Annexin V-FITC and 5 *μ*L PI (50 *μ*M) working solution were added to every 200 *μ*L of cell suspension. The cells were incubated at room temperature for 20 min in the dark and analyzed by flow cytometry. The Annexin V and PI emissions were detected in the FL1-H and FL2-H channels of a FACSVantage flow cytometer, using emission filters of 525 and 575 nm, respectively. The results were shown as quadrant dot plots with intact cells (Annextin V−/PI−), early apoptotic cells (Annextin V+/PI−), late apoptotic cells (Annextin V+/PI+), and necrotic cells (Annextin V−/PI+). The number of each kind of cells was expressed as percentages of the number of total stained cells. Data were acquired using Cellqest software and analyzed by ModFit software.

### 2.9. Isolation of Mitochondrial and Cytosolic Fractions and Western Blotting Analysis for Detection of Cyt-C and AIF

Cell cytosolic and mitochondrial fractionations were performed as described [47, 48]. Briefly, HepG2 cells (3 to 3.6 × 10^6^) were homogenized using a Dounce homogenizer on ice in a homogenization buffer [10 mM HEPES, pH 7.4, 250 mM sucrose, 10 mM KCl, 1.5 mM MgCl2, 1 mM EDTA, 1 mM EGTA, 1 mM DTT plus protease inhibitor cocktail (Roche Molecular Biochemicals)], followed by 2000 ×g centrifugation for 5 minutes at 4°C; the supernatant was centrifuged at 15,000 ×g for 25 minutes at 4°C and used as the cytosolic component containing the released Cyt-C. The pellets were lysed with RIPA buffer for 10 minutes on ice, and supernatants were added to the sample buffer to obtain the mitochondria fraction. Protein concentration was determined by Lowry's method. Western blot was performed to identify the released Cyt-C and AIF. *β*-actin and COX-IV were used as cytosolic and mitochondrial component loading controls, respectively. Next, proteins (50 *μ*g/sample) were run on 15% SDS-PAGE and transferred to PVDF membranes. The membranes were blocked with 5% nonfat milk in Tris-buffered saline and incubated with antibody to cleaved Cyt-C (1 : 500), AIF (1 : 500), *β*-actin (1 : 5000), and COX-IV (1 : 750), respectively. The reaction was followed by the addition of a horseradish-conjugated secondary anti-mouse antibody (1 : 2000) or anti-rabbit antibody (1 : 2000), and bands were visualized with an ECL system according to the manufacturer's instructions. Densitometric analyses of the western blot bands were performed using optical density scanning and ImageJ software. The ratio of target protein optical density values to those of the internal reference protein *β*-actin or COX-IV was determined for each group.

### 2.10. Western Blotting Analysis for Detection Activities of Caspase-9 and Caspase-3

HepG2 cells were exposed to 200 *μ*M H_2_O_2_ with or without NAS. Cells were collected in lysis buffer [20 mM Tris, pH 8.0, 137 mM NaCl, 10% glycerol/1% Nonidet P-40, 2 mM EDTA with 5 mM Na2VO4, protease inhibitor mixture (Roche Molecular Biochemicals), 0.2 mM phenylmethylsulfonyl fluoride] on ice, centrifuged at 19,720 ×g for 10 minutes at 4°C, cytoplasmic proteins were extracted, and protein concentration was determined by Lowry's method. Samples of total protein lysates (50 *μ*g/sample) were run on 10% SDS-PAGE gels and caspase-9 and caspase-3 were analyzed by western blot.

### 2.11. Immunocytochemistry

Cultured HepG2 cells were exposed to H_2_O_2_ with or without 10 *μ*M NAS and then fixed in 4% paraformaldehyde for 15 minutes and permeabilized with 0.05% Triton X-100. After blocking with 5% BSA, cells were incubated with antibody to cleaved caspase-3 (1 : 200), with cleaved caspase-9 (1 : 200), and incubated with FITC-conjugated secondary antibodies (1 : 150), and these fluorescent slices were counterstained with Hoechst 33342 (1 : 10,000) for 5 minutes. These incubations were conducted in the dark so as not to bleach the fluorescent labels. As negative controls, we performed staining in the absence of each of the primary antibodies and added bovine serum albumin. Images were taken using a fluorescence microscope (OLYMPUS, BX53F).

### 2.12. Rhodamine 123 Staining

HepG2 cells were cultured in 6-well plates and pretreated with vehicle or 10 *μ*M NAS for 2 hours and then treated with 200 *μ*M H_2_O_2_ for 6 hours. Mitochondrial membrane potential was determined using rhodamine 123, a cationic fluorescent indicator that selectively accumulates within mitochondria in a membrane potential depending manner, as described previously [49, 50]. Briefly, the cultured cells were directly incubated with 2 *μ*M rhodamine 123 for 25 minutes at room temperature in the dark, followed by rinsing with several changes of PBS (5 minutes per rinse). Images were taken using a fluorescence microscope (OLYMPUS, BX53F). A reduction in green rhodamine 123 fluorescence indicates dissipated ΔΨ_*m*_.

Quantification of mitochondrial transmembrane potential (ΔΨ_*m*_) was determined using tetramethyl rhodamine methyl ester (TMRM), a lipophilic cation that selectively accumulates within mitochondria in a membrane potential depending manner. Briefly, the cells from different groups were trypsinized following a wash in PBS. Then, add 15 *μ*L of TMRM working solution to every 1000 *μ*L of cell suspension (final concentration 150 nM of TMRM) for 25 minutes at 37°C, allowing mitochondria to load with TMRM fluorescent dye in proportion to mitochondrial membrane potential. Following washing 3 times with PBS gently, TMRM fluorescence was detected on a FACSCalibur flow cytometer.

### 2.13. Statistical Analysis

All data are expressed as mean ± standard deviation (SD) and were analyzed using SPSS 11.0 software (SPSS, Chicago, IL). *P* < 0.05 was considered statistically significant.

## 3. Results 

### 3.1. NAS Inhibits H_2_O_2_-Induced Cell Death of HepG2 Cells

**Figure 1:**
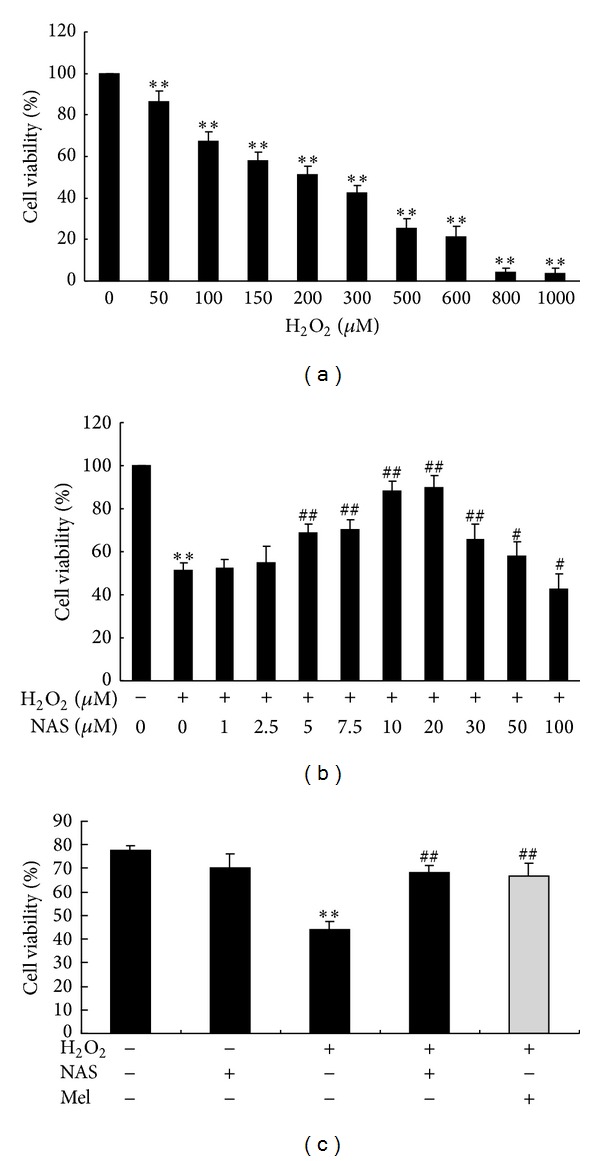
Cell viability of HepG2 cells assayed by MTT. The results are expressed as percentage of control, and each value represents the mean ± SD of six independent experiments. The annotation **indicates a *P* value < 0.01 versus untreated group. The annotation ^#^indicates a *P* value < 0.05 versus H_2_O_2_ group. The annotation ^##^indicates a *P* value < 0.01 versus H_2_O_2_ group. (a) Cell viability of HepG2 cells following different concentrations of H_2_O_2_ exposure were measured by MTT assay. (b) Cytotoxicity of NAS to HepG2 cells was measured by MTT assay. HepG2 cells were treated by 200 *μ*M H_2_O_2_ with or without a series of concentrations of NAS (1, 2.5, 5, 7.5, 10, 20, 30, 50, and 100 *μ*M) for 6 hours. (c) The effect of NAS on HepG2 cell viability in response to H_2_O_2_ and comparing it with melatonin (grey bar), a well standardized antioxidant.

H_2_O_2_ has often been used in the oxidative stress injury model with hepatocytes as well as other cell types [17–22]. To determine the proper working concentrations of H_2_O_2_, we performed a series of dose-response assays. HepG2 cells were randomly assigned to nine groups: the untreated group and H_2_O_2_-treated groups with different concentrations (50, 100, 150, 200, 300, 500, 600, 800, or 1000 *μ*M) for 6 hours. The viability of HepG2 cells in different groups was measured by MTT assay. We found that 200 *μ*M H_2_O_2_ caused cell viability to decrease by about 50% (Figure 1(a)). Therefore we exposed HepG2 cells to concentration of 200 *μ*M H_2_O_2_ for 6 hours to establish an oxidative stress injury model. To evaluate the most effective concentrations of NAS, H_2_O_2_-induced HepG2 cells were treated with different concentrations of NAS (from 1 to 100 *μ*M). We found that NAS showed protective effects in a dose-dependent manner in the range 2.5–10 *μ*M. There was no difference between 10 and 20 *μ*M. Cell viability in the 30- and 100-*μ*M groups was significantly lower than in the 20-*μ*M group (Figure 1(b)). Therefore we used 10 *μ*M in this study. As shown in Figure 1(c), the cell viability of HepG2 cells was significantly decreased after treatment with 200 *μ*M H_2_O_2_ (*n* = 6, *P* < 0.01). However, NAS treatment effectively protected HepG2 cells from H_2_O_2_-induced cell death (*n* = 6, *P* < 0.01). The difference was statistically significant (*P* < 0.01). While NAS alone has no effect on the cell viability of HepG2 cells (Figure 1(c)), we further compared the effect of NAS with another well standardized antioxidant melatonin on cell viability of HepG2 cells and found that there was no difference between H_2_O_2_ + NAS group and H_2_O_2_ + melatonin group. The data indicate that NAS, similar with melatonin does, effectively protects HepG2 cells from H_2_O_2_-induced cell death.

### 3.2. NAS Inhibits H_2_O_2_-Induced Reactive Oxygen Species Production in HepG2 Cells

**Figure 2:**
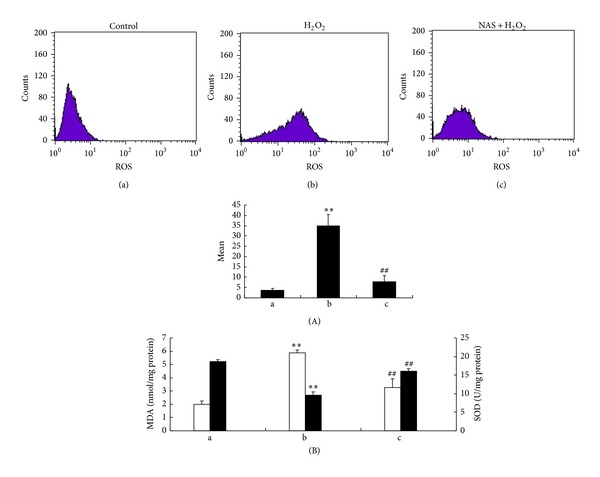
NAS reduces H_2_O_2_-induced reactive oxygen species production. (A) Intracellular ROSdetected by flow cytometry afterH2DCF staining. (B) MDA content (white bars) and SOD activity (black bars) were measured in HepG2 cell lysates. (a) Untreated group; (b) H_2_O_2_ group, HepG2 cells incubated with 200 *μ*M H_2_O_2_ for 6 hours; (c) H_2_O_2_ + NAS group, 10 *μ*M NAS was administered before subjecting to H_2_O_2_. The significant increase in MDA levels and decrease in SOD levels in the H_2_O_2_ group compared with untreated group are shown (*P* < 0.01). NAS attenuated the H_2_O_2_-induced changes in MDA and SOD (*P* < 0.01). Error bars represent SD (*n* = 3). The annotation **indicates a *P* value < 0.01 versus untreated group. The annotation ^##^indicates a *P* value < 0.01 versus H_2_O_2_ group. Experiments were performed at least three times.

To determine the effect of NAS on H_2_O_2_-induced intracellular ROS production, the intracellular ROS was visualized by detecting dichlorofluorescein (DCF) derived from the oxidation of H2DCF. The results show that untreated HepG2 cells had little basal intracellular ROS. However, after exposure to H_2_O_2_, cells had significantly increased intracellular ROS accumulation (*P* < 0.05). The intensity of the mean oxidized DCF in untreated and H_2_O_2_ group was (3.60 ± 1.02) and (34.85 ± 5.67), respectively. The intensity in NAS-treated group was (7.85 ± 2.93), which was significantly lower than in the H_2_O_2_ group. To determine whether the cytoprotective effect of NAS is a consequence of the breakdown of the endogenous antioxidant defense mechanisms, we investigated the levels of MDA, an end product of lipid peroxidation, and SOD, an oxygen radical scavenger [51]. As demonstrated in Figure 2, incubation of HepG2 cells with H_2_O_2_ caused a significant increase in MDA, intracellular ROS, and a marked decrease in SOD activity compared with untreated cells (*P* < 0.05). However, NAS significantly attenuated those changes of MDA, intracellular ROS, and SOD (*P* < 0.05). This observation suggests that H_2_O_2_ could induce ROS accumulation in HepG2 cells by breakdown of the balance of the endogenous antioxidant defense mechanisms and NAS effectively reduces H_2_O_2_-induced ROS production.

### 3.3. NAS Inhibits H_2_O_2_-Induced HepG2 Cell Apoptosis

**Figure 3:**
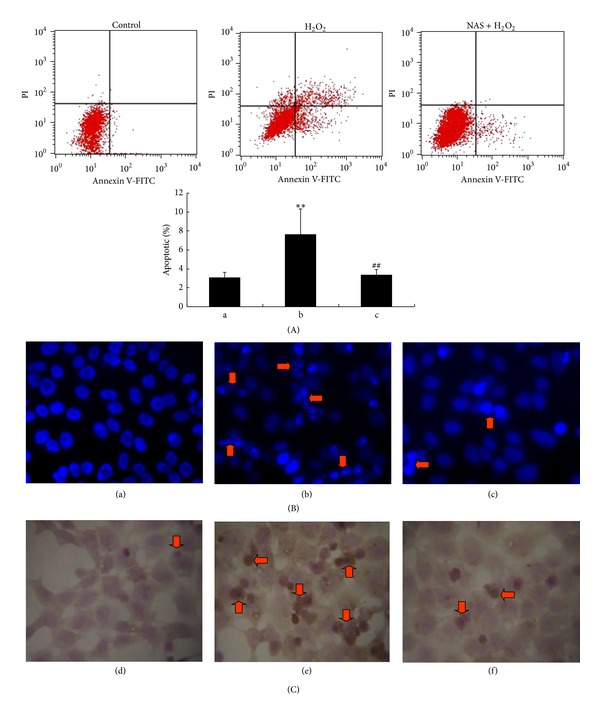
NAS inhibits H_2_O_2_-induced HepG2 cells apoptosis. (A) Apoptotic cells detected by flow cytometry after annexin V and PI double-staining. (B) (a, b, c) Morphologic changes in nuclei observed with Hoechst 33342 staining under fluorescence microscopy. (C) (d, e, f) Apoptotic cells detected by TUNEL staining. (a, d) Untreated group; (b, e) H_2_O_2_ group, HepG2 cells incubated with 200 *μ*M H_2_O_2_ for 6 hours; (c, f) H_2_O_2_ + NAS group, 10 *μ*M NAS was administered before subjecting to H_2_O_2_. Note that untreated group cells appeared to have a normal shape with a round intact nuclei; H_2_O_2_-treated cells showed reduced nuclear size, strong fluorescent spot, and pyknotic nuclei, indicating apoptotic nuclei; the strong fluorescent spots were almost completely abrogated by pretreatment with NAS. The arrows indicate apoptotic cells. Original magnification ×400.

To evaluate the cytoprotective effect of NAS on H_2_O_2_-induced HepG2 cell apoptosis, three assays (Annexin V and PI double-staining, Hoechst 33342 staining and TUNEL staining) were conducted. The results of Annexin V and PI double staining demonstrated that an increase of apoptotic cells was observed in H_2_O_2_-treated group with a lower number of living cells. The apoptotic percentage was (3.07 ± 0.57)% in the untreated group, which was significantly lower than in the H_2_O_2_ group (7.61 ± 2.73)% (*P* < 0.01). Compared to H_2_O_2_ group, the apoptotic percentage in NAS-treated group (3.37 ± 0.56)% was decreased significantly (*P* < 0.01). The HepG2 cells in the untreated group showed normal shape with round intact nuclei (Figures 3(a) and 3(d)), whereas the H_2_O_2_-treated cells became more scarce and showed reduced nuclear size, extensive blebbing, strong fluorescent spot, and pyknotic nuclei (Figures 3(b) and 3(e)), indicating condensed chromatin and apoptotic bodies. In agreement with the results of Hoechst 33342 staining, there were occasional TUNEL-positive cells in the untreated group untreated group. Compared to the untreated group, the H_2_O_2_ group had significantly more TUNEL-positive cells. However, those changes in HepG2 cells were abrogated significantly by pretreatment with NAS (Figures 3(c) and 3(f)).

### 3.4. NAS Inhibits H_2_O_2_-Induced Dissipation of ΔΨ_*m*_ of HepG2 Cells

**Figure 4:**
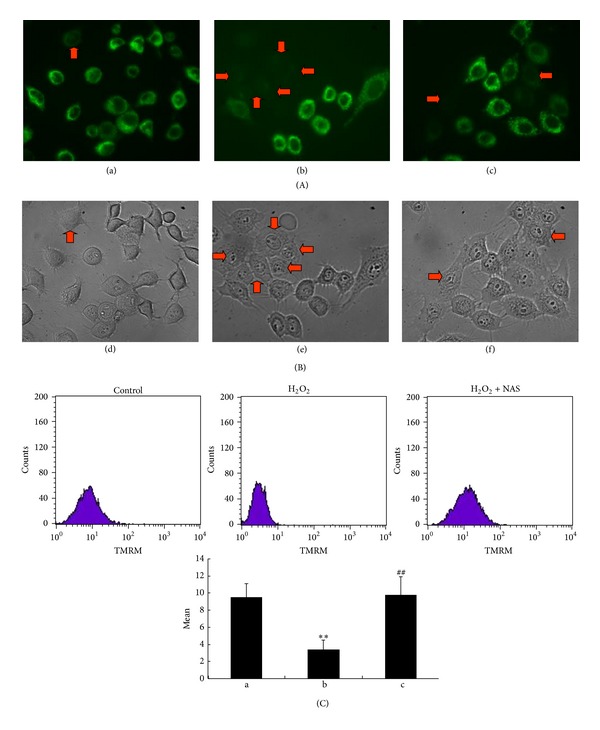
NAS inhibits the dissipation of ΔΨ_*m*_ of HepG2 cells induced by H_2_O_2_. (A) (a, b, c): ΔΨ_*m*_ observed with rhodamine 123 staining under fluorescence microscopy. (B) (d, e, f) Morphologic changes in nuclei observed under the inverted phase contrast microscope. (a, d) Untreated group; (b, e) H_2_O_2_ group, HepG2 cells incubated with 200 *μ*M H_2_O_2_ for 6 hours; (c, f) H_2_O_2_ + NAS group, 10 *μ*M NAS was administered before subjecting to H_2_O_2_. (C) ΔΨ_*m*_ detected by flow cytometry after TMRM staining. Note that untreated group cells display a granular pattern of TMRM fluorescence in cytoplasm; H_2_O_2_-treated cells showed diffuse pattern of TMRM fluorescence, indicating apoptotic nuclei; the dissipation of ΔΨ_*m*_ was almost completely abrogated by pretreatment with NAS. The arrows indicate the apoptotic cell. Original magnification ×400.

Proper ΔΨ_*m*_, the charge difference across a membrane that is permeable to ions, is critical for cellular bioenergetic homeostasis, and collapse of the ΔΨ_*m*_ is an important event associated with mitochondrial dysfunction, or even cell death [52]. Thus the ΔΨ_*m*_ assay was used as a more specific test for early mitochondrial injury. Both rhodamine 123 and TMRM are cationic fluorescent dye, which could enter the mitochondrial matrix and cause photoluminescent quenching dependent on ΔΨ_*m*_. We therefore investigated possible loss of ΔΨ_*m*_ using rhodamine 123 and TMRM staining. The results showed that rhodamine 123 fluorescence in untreated group cells displayed a punctate staining pattern within the cell (Figures 4(a) and 4(d)). Following treatment with H_2_O_2_ for 6 hours, rhodamine 123 fluorescence became diffuse because of mitochondrial depolarization (Figures 4(b) and 4(e)), and NAS treatment preserved the punctate staining pattern of rhodamine 123 distribution in the presence of a cellular stress (Figures 4(c) and 4(f)). As shown in Figure 4(c), the fluorescence intensity of TMRM in H_2_O_2_ group decreased significantly compared to the untreated group. Cells of NAS group exhibited much more TMRM fluorescence intensity than H_2_O_2_ group. These data provide evidence that NAS-mediated protective effects not only involve rescue of nuclear morphology changes, but also prevent the dissipation of ΔΨ_*m*_ induced by H_2_O_2_.

### 3.5. NAS Inhibits the Release of Mitochondrial Apoptogenic Factors from Mitochondria Induced by H_2_O_2_

**Figure 5:**
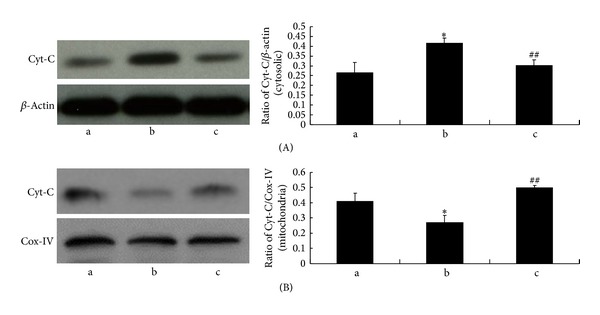
NAS reduces the release of Cyt-C from mitochondria in response to H_2_O_2_. The cells were treated with 200 *μ*M H_2_O_2_ for 6 hours with or without NAS. Cytosolic and mitochondrial fractions were prepared, and the expression of Cyt-C was determined by western blot analysis. (A) Cytosolic fractions. (B) Mitochondrial fractions. (a) Untreated group; (b) H_2_O_2_ group, HepG2 cells incubated with 200 *μ*M H_2_O_2_ for 6 hours; (c) H_2_O_2_ + NAS group, 10 *μ*M NAS was administered before subjecting to H_2_O_2_. Note the significantly increased Cyt-C in cytosolic and decreased Cyt-C in mitochondria in H_2_O_2_ group compared with untreated group (*P* < 0.01). NAS attenuated the changes in Cyt-C. The results are expressed as ratio of Cyt-C optical density values to those of *β*-actin or COX-IV, and each value represents the mean ± SD of three independent experiments. The annotation *indicates a *P* value < 0.05 versus untreated group. The annotation ^##^indicates a *P* value < 0.01 versus H_2_O_2_ group.

**Figure 6:**
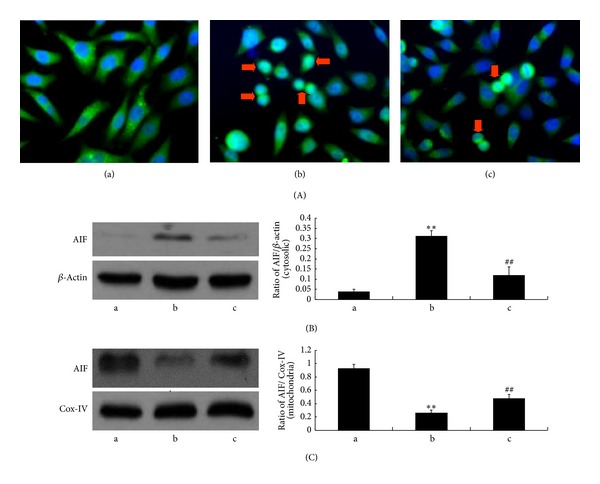
NAS inhibits the release of AIF from mitochondria in response to H_2_O_2_. (a) Untreated group; (b): H_2_O_2_ group, HepG2 cells incubated with 200 *μ*M H_2_O_2_ for 6 hours; (c) H_2_O_2_ + NAS group, 10 *μ*M NAS was administered before subjecting to H_2_O_2_. (A) The expression of AIF as assessed by immunocytochemistry ((a)–(c): 400x). (B, C): Cytosolic and mitochondrial fractions were prepared, and the expression of AIF was determined by western blot analysis. (B) Cytosolic fractions; (C) mitochondrial fractions. Note the AIF nuclear translocation and significantly increased AIF in cytosolic fractions and decreased AIF in mitochondrial fractions in the H_2_O_2_ group compared with untreated group (*P* < 0.01). NAS attenuated the changes in AIF. The results are expressed as ratio of AIF optical density values to those of *β*-actin or COX-IV, and each value represents the mean ± SD of three independent experiments. The annotation *indicates a *P* value < 0.05 versus untreated group. The annotation ^##^indicates a *P* value < 0.01 versus H_2_O_2_ group.

Release of mitochondrial apoptogenic factors from the mitochondria into the cytoplasm is a common feature of cell death. We and other researchers previously reported that NAS effectively inhibited the release of Cyt-C and AIF from the mitochondria into the cytoplasm in apoptotic cells under oxidative stress injury induced by H_2_O_2_ and melatonin [35], while melatonin protected AIF-dependent cell death in a model of acute liver failure through its direct inhibition of hepatic RIP1 and subsequent JNK phosphorylation [53]. We therefore investigated the expression of Cyt-C and AIF in the cytosolic fractions and mitochondrial fractions of hepG2 cells by western blot analysis. As shown in Figures 5 and 6, H_2_O_2_ induced an increase of Cyt-C and AIF in the cytosolic fractions and a decrease in the mitochondrial fractions (*P* < 0.01). NAS effectively blocked leakage of AIF induced by H_2_O_2_ (*P* < 0.01).

### 3.6. NAS Inhibits Activation of Caspases Induced by H_2_O_2_ in HepG2 Cells

**Figure 7:**
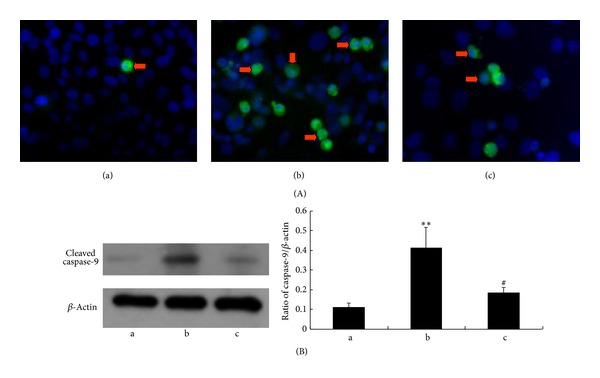
NAS prevents the activation of caspase-9. (A) Activities of caspase-9 as assessed by immunocytochemistry (400x). Cells were immunostained with active-caspase-9 antibody and stained with DAPI. (B) Activities of caspase-9 as assessed by western blot. (a) Untreated group; (b) H_2_O_2_ group, HepG2 cells incubated with 200 *μ*M H_2_O_2_ for 6 hours; (c) H_2_O_2_ + NAS group, 10 *μ*M NAS was administered before subjecting to H_2_O_2_. Note the increased activities of caspase-9 in the H_2_O_2_ group compared with the untreated group (*P* < 0.01) and the decreased activities in the H_2_O_2_ + NAS group compared with the H_2_O_2_ group (*P* < 0.01). The results are expressed as ratio of cleaved caspase-9 optical density values to those of *β*-actin, and each value represents the mean ± SD of three independent experiments. The annotation **indicates a *P* value <0.01 versus untreated group. The annotation ^#^indicates a *P* value < 0.05 versus H_2_O_2_ group.

**Figure 8:**
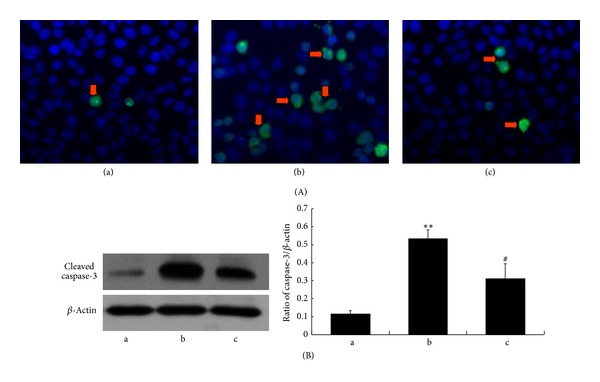
NAS prevents the activation of caspase-3. (A) Activities of caspase-3 as assessed by immunocytochemistry ((a)–(c): 400x). HepG2 cells were immunostained with active-caspase-3 antibody and FITC-conjugated secondary antibody and then stained with DAPI. (B) Activities of caspase-3 as assessed by western blot. (a) Untreated group; (b) H_2_O_2_ group, HepG2 cells incubated with 200 *μ*M H_2_O_2_ for 6 hours; (c) H_2_O_2_ + NAS, 10 *μ*M NAS was administered before exposure to H_2_O_2_. Note the activities of caspase-3 in the H_2_O_2_ group compared with the untreated group (*P* < 0.01) and the decreased activities in the H_2_O_2_ + NAS group compared with the H_2_O_2_ group (*P* < 0.01). The results are expressed as ratio of cleaved caspase-3 optical density values to those of *β*-actin, and each value represents the mean ± SD of three independent experiments. The annotation **indicates a *P* value < 0.01 versus untreated group. The annotation ^#^indicates a *P* value < 0.05 versus H_2_O_2_ group.

Once Cyt-C and AIF trigger cytosolic reaction cascades encompassing caspases and regulatory factors, which eventually lead to caspase-dependent and caspase-independent cell death. As an important modulator of cell death, caspase activation was evaluated in our study. Because we were most interested in evaluating mitochondrial-dependent pathway events, we measured the activities of caspases-9 and caspase-3 by immunocytochemistry and western blot. As shown in Figures 7 and 8, caspase-9 and caspase-3 were activated by H_2_O_2_ insult, and NAS effectively inhibited their activation. These results, together with its ability to attenuate the release of Cyt-C and AIF from mitochondria, demonstrate that NAS prevents oxidant-induced apoptosis through inhibition of mitochondrial-dependent cell death pathways.

## 4. Discussion

Hepatic I/R injury occurs in liver transplantation, hepatic resection, abdominal surgery with hepatic vascular occlusion, and coronary bypass surgery [54–59], which is a major cause of primary nonfunctioning graft. Oxidative stress associated with the formation of ROS plays an important role in the pathogenesis of hepatic I/R injury. As the primary cellular consumer of oxygen, together with multiple electron carriers and redox enzymes, mitochondria are considered a main source of ROS. However, mitochondria not only produce ROS, but are also targets of oxidative stress. In this way ROS-induced ROS release contributes to oxidative damage and mitochondrial dysfunction in a range of diseases.

Mitochondria not only play a crucial role in energy generation and intermediary metabolism, but are also known as gatekeepers in the intrinsic pathway or mitochondrial-mediated apoptosis [60]. Activation of the intrinsic pathway involves release of proapoptotic factors like Cyt-C, Smac/Diablo, Endonuclease G, and AIF from the mitochondrial intermembrane space into the cytosol. Among proapoptotic factors, Cyt-C has been the most intensively studied. Once released into the cytosol, Cyt-C triggers the formation of the apoptosome, an oligomeric complex of Cyt-C/Apaf-1/caspase-9, whose function is the recruitment and activation of caspase 9, which is the “initiator” caspase [61]. The cleaved caspase-9 targets and activates caspase-3, which is the “executioner” or “effector” caspases, catalyzing the final steps of the apoptotic signaling cascade [62]. Based on the important role of oxidative damage and mitochondrial dysfunction in a range of diseases, antioxidant and mitochondria-protective strategies will be of great importance in counteracting oxidative stress injury.

NAS is the immediate precursor of melatonin, the pineal gland indole. There are chemical and biological similarity between NAS and melatonin. Both of NAS and melatonin have the common indole ring, while hydroxy group of NAS is replaced by a methoxy at the same position of melatonin in the indole ring. Many of the biological effects of NAS including neuroprotection, antioxidant, circadian rhythm and sleep adjustment, antidepressant, antianxiety, antiaging, analgesic effects, and lowering blood pressure are similar to those produced by melatonin [28, 32, 35, 63, 64]. However, in-depth research eventually revealed that NAS has its own biological properties including its action as a potent TrkB receptor agonist and NAS is found in some areas of the brain where melatonin is absent [65]. In recent years, NAS has been reported to exert significant antioxidant properties both* in vitro* and* in vivo*. Using an erythrocyte oxidative damage model induced by cumene hydroperoxide and H_2_O_2_, Barsacchi found that 100–400 *μ*M NAS, but not melatonin, has dose-dependent antioxidant effects [66]. Leaden and Catalá [67] and García et al. [68] found that NAS strengthened biological membranes against oxidative stress, which may be related to its ability to reduce lipid peroxidation. Additional studies have shown that NAS also exerts protective effects against peroxidative damage of neurons [69–71], lung epithelial cells [72], erythrocytes [73], testicular cells [74], retinal cells [75, 76], and lymphocytes [77]. The data imply that the antioxidant effect of NAS is independent of melatonin and stronger than that of melatonin [76, 78]. However, little is known about the effect of NAS on hepatic I/R injury.

In the present study, we show that H_2_O_2_ markedly decreases the viability of HepG2 cells, whereas pretreatment with NAS significantly inhibits cell injury, as demonstrated by MTT assay (Figure 1(c)). Our results indicate that while H_2_O_2_ can cause HepG2 cell death, NAS pretreatment effectively protects HepG2 cells from H_2_O_2_-induced damage.

Oxidative stress caused by ROS is responsible for a wide variety of cellular damage and is the most validated mechanism of secondary injury [79]. Following oxidative stress, the overproduction of ROS and subsequently the depletion of antioxidants resulted in the total breakdown of the endogenous antioxidant defense mechanisms, culminating in failure to protect cells from oxidative damage. Among biomarkers of oxidative stress, MDA and SOD are known as two sensitive indicators [48]. MDA is the end product of lipid peroxidation [80] and MDA levels reflect the extent of cell damage due to oxidative stress. SOD is an oxygen radical scavenger that scavenges superoxide radicals by converting them to hydrogen peroxide, which is then converted to water by catalase and GSH-Px [81]. In the present study, increased intracellular ROS and MDA levels and decreased SOD activity were noted in H_2_O_2_-induced HepG2 cells, suggesting that oxidative stress caused by ROS is involved in the pathogenesis of H_2_O_2_-induced HepG2 cellular injury. However, NAS reduced the decline in SOD activity and augmented MDA content (Figure 2). Our study demonstrates that NAS treatment significantly alleviates oxidative stress by reducing levels of ROS and MDA and increasing SOD activity in H_2_O_2_-induced HepG2 cells.

Through a variety of studies, we know that oxidants can not only stimulate inflammatory cytokine such as interleukin-8 [37, 82] in HepG2 cells and cause cellular senescence [83], but also induce apoptosis in HepG2 cells, [37]; certain apoptotic agents increase the production of ROS in mitochondria [84], and antioxidants such as N-acetylcysteine [85, 86], melatonin [87, 88], NAS [26], and Vitamin E [27] can prevent apoptosis. Such studies also confirmed the association between ROS-apoptosis. Several studies have shown that the level of apoptotic hepatocytes during hypoxia mirrored the level of intracellular ROS production. During the process of oxidative stress injury, mitochondria are both a significant source of ROS and also a target of damage, with a variety of consequences. Based on the prominent role of mitochondria in oxidative stress injury, many studies have clearly shown that ROS leads to cell apoptosis via the mitochondria-dependent apoptotic pathway, which involves damage to the mitochondrial membrane, release of Cyt-C into the cytoplasm followed by caspase-3 activation, and finally hepatocyte apoptosis [30]. The morphologic changes in nuclei, the dissipation of ΔΨ_*m*_, the release of Cyt-C, and the expression of cleaved caspase-3 were employed as apoptotic biomarkers to elucidate the mechanisms underlying the antiapoptotic effects of NAS.

ΔΨ_*m*_ is a sensitive indicator of mitochondrial function. The decline of mitochondrial transmembrane potential leads to the release of caspase-activating proteins [89]. Increasing evidence suggests that the ΔΨ_*m*_ assay can be used as a more specific test for early mitochondrial injury. In this study, H_2_O_2_ group showed a significant decrease in mitochondrial membrane potential. Interestingly, NAS treatment preserved mitochondrial membrane potential, suggesting the inhibition of mitochondrial membrane permeability.

ROS can also induce the release of apoptogenic factors from mitochondria, which can trigger caspase cascade. Caspases are a family of cysteine proteases that cleave target proteins at specific residues. Among the more than ten members of the caspase family identified, the extensively studied caspase-3 (the “executor of apoptosis,”) plays a crucial role in cell death [90, 91]. In this study, upregulated cleaved caspase-9 and caspase-3, together with significantly decreased expression of Cyt-C and AIF in mitochondria and increased expression in the cytosol of H_2_O_2_-induced cells, indicate that the cells underwent apoptosis by a mitochondrial-dependent pathway; these parameters were lowered in the group pretreated with NAS. These findings imply that the ability of NAS to attenuate oxidative stress partly depends on inhibiting mitochondrial-related apoptosis.

In summary, the present study shows that H_2_O_2_ can damage HepG2 cells and cause cells apoptosis. NAS protects human HepG2 cells against H_2_O_2_-induced oxidative stress, as measured by cell viability, cell apoptosis, ROS activity, the dissipation of ΔΨ_*m*_, release of mitochondrial apoptogenic factors, and activities of caspase-9 and caspase-3. NAS-mediated protection can be conferred by one or more of the following mechanisms: first, NAS could reduce the oxidative stress injury by restoration of endogenous antioxidation and the decrease of lipid peroxidation. Second, NAS could attenuate apoptosis through inhibiting the subsequent biochemical changes in the mitochondria-dependent apoptotic pathway, such as release of mitochondrial apoptogenic factors, activation of caspase family members, and alteration of mitochondrial membrane permeability. These data help explain the protective action of NAS against cell injuries involving the mitochondrial pathway.
